# Supplementary material for: Uridine Prevents Negative Effects of OXPHOS Xenobiotics on Dopaminergic Neuronal Differentiation
Source: Cells. 2019 Nov 8;8(11):1407. doi: 10.3390/cells8111407 (PMC6912777; doi:10.3390/cells8111407)
Supplement: Supplementary file 1 [file cells-08-01407-s001.pdf]

SUPPLEMENTAL NOTE 1- Dopaminergic neuronal differentiation

Cell model genetic characterization. The neuroblastoma SH-SY5Y cell line has been frequently used for PD study [1,2]. In fact, by considering altogether “SH-SY5Y” and “Parkinson’s” terms, more than 1,300 publications appear in the PubMed database. Because cross-contamination of human cell lines with other cell lines is a widely acknowledged issue [3], we determined the nuclear genetic fingerprint of this cell line based on 16 short tandem repeats (STRs) and confirmed its identity (Table S1) [4].

**CHR**	2	2	3	4	5	5	7	8
**STR**	D2S1338	TPOX	D3S1358	FGA	CSF1PO	D5S818	D7S820	D8S1179
**SH-SY5Y**	17, 19	8, 11	15, 16	23.2, 24	11	12	7, 10	15
**SH-SY5 (ATCC)**		8, 11			11	12	7, 10	
**CHR**	11	12	13	16	18	19	21	X
**STR**	TH01	vWA	D13S317	D16S539	D18S51	D19S433	D21S11	AMEL
**SH-SY5Y**	7, 10	14, 18	11	8, 13	13, 16	13,14	31, 31.2	X
**SH-SY5 (ATCC)**	7, 10	14, 18	11	8, 13				X

Table S1. Nuclear genetic fingerprint of human neuroblastoma SH-SY5Y cell line. CHR, chromosome; STR, short tandem repeat; ATCC, American type culture collection (https://www.lgcstandards-atcc.org/?geo_country=es).

Growth conditions can induce changes in the cell line karyotype during long-term culture [5]. To check the chromosomal stability of our cell lines and, therefore, their suitability as cell models, a karyotype analysis was carried out. The karyotype of neuroblastoma SH-SY5Y cells was 47, XX, +7 (Figure S1A). These cells showed the same chromosomal modal number and abnormalities than those previously reported [6-11]. For human neural stem cells (hNSCs), one out of 20 metaphases showed a trisomy 20 but the rest were euploids (Figure S1B). This trisomy is a relatively common and recurrent chromosomal abnormality [12]. Despite of this, the results suggest that these cell lines are stable enough from a karyotype point of view to consider them as good cell models.

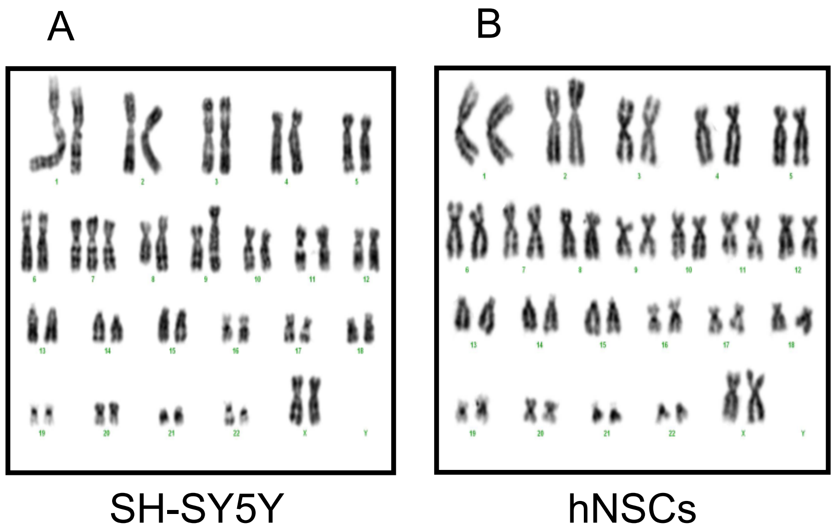

Figure S1. Karyotypes. A) Neuroblastoma SH-SY5Y cell line. The chromosomal modal number and range was 47 and 46-48, respectively. Chromosomal abnormalities were: ins(1q), +7, ?der(8)(q24), add(9)(q34?), der(22)add(22)(q13). B) Human neural stem cells (hNSCs). The chromosomal modal number and range was 46 and 46-47, respectively. A trisomy in chromosome 20 was found in one metaphase.

To characterize their mtDNA genotypes, we sequenced them. hNSCs belong to mtDNA haplogroup K1a1b1a (GenBank KX350098.1) [13]. Haplogroup K has been proposed as a protective mtDNA genetic background against PD [14]. The absence of m.7028C>T transition in SH-SY5Y cells suggested its phylogenetic origin inside mtDNA genetic background H [15]. The mtDNA sequences of SH-SY5Y cells classified them as belonging to mtDNA haplogroup H48 (GenBank KX350100.2). It has been proposed that haplogroup H increases the risk for PD [14]. The mtDNA sequences did not harbor pathologic mutations [16,17]. Finally, making neuroblastoma SH-SY5Y cell line a more interesting model for PD, its whole genome has been re-sequenced and most of the genes belonging to the major PD pathways were intact [18].

Cell differentiation into dopaminergic neuron. Population doubling times of SH-SY5Y cell line in high glucose (25 mM) DMEM and hNSCs in KnockOut DMEM F-12 (glucose 17.5 mM) are 33.8 h and 45.8 h, respectively.

After only two days, differentiation protocols in 5 mM galactose increase cell processes (neurites) number and length (Figure S2). At the end of these protocols, 70-90 % SH-SY5Y cells and 55-80 % hNSCs were morphologically differentiated.

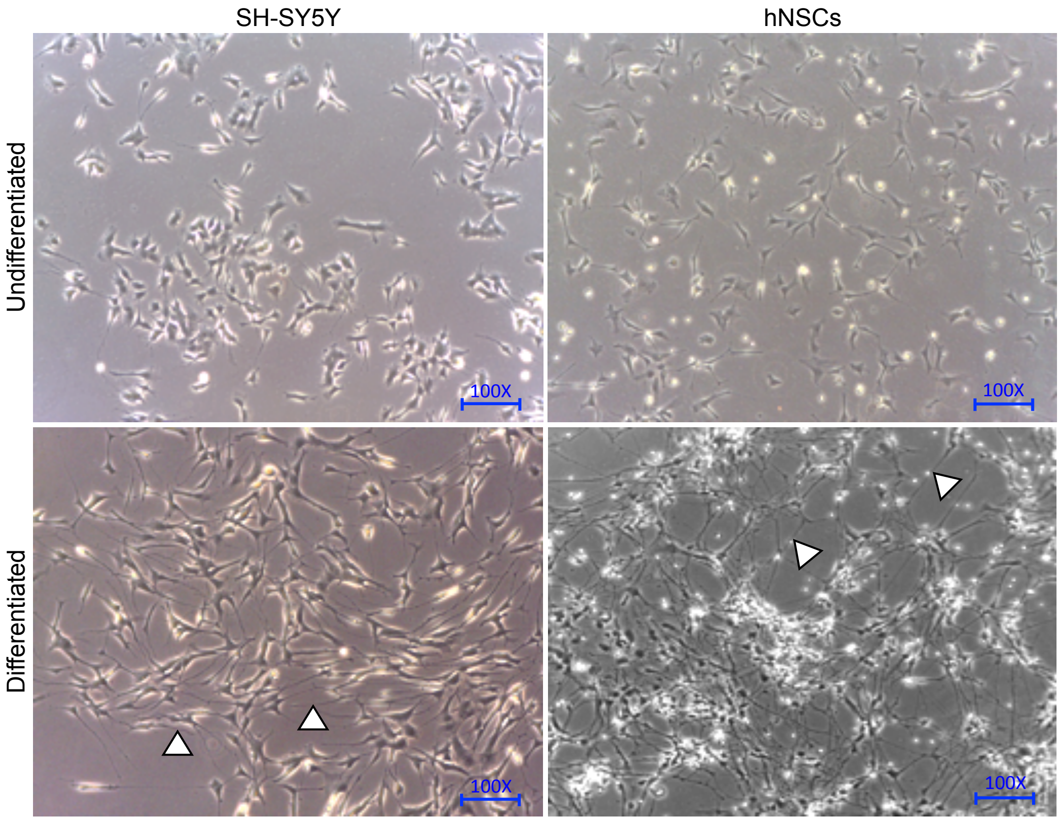

Figure S2. Undifferentiated and differentiated neuroblastoma SH-SY5Y cells and hNSCs. Neurites are indicated (arrowheads) in differentiated cells.

Neurites generation requires cytoskeleton remodeling and the levels of cytoskeletal related proteins βIII-tubulin (TUBB3), doublecortin (DCX), microtubule-associated protein 2 (MAP2) and nestin (NES) are commonly used as markers for neuronal differentiation [19,20]. The presence of the glycolytic isoenzyme neuron-specific enolase (NSE) is a late event in neuronal differentiation [21,22]. Flow cytometry (FC) analysis shows that NES levels decrease in hNSCs. However, and similar to other report [23], they increase in the SH-SY5Y cell line after our differentiation protocol (Figure S3A). TUBB3 and DCX levels increase after the differentiation in both neuroblastoma cell line and hNSCs. These results suggest that SH-SY5Y cell populations at different stages of differentiation are represented in the differentiated cultures: undifferentiated cells, partially differentiated NES+ cells, and neuron-like cells. Western blot (WB) analyses confirm the increase of TUBB3 and DCX, and 2 other, neural markers, such as MAP2 and NSE (Figure S3B).

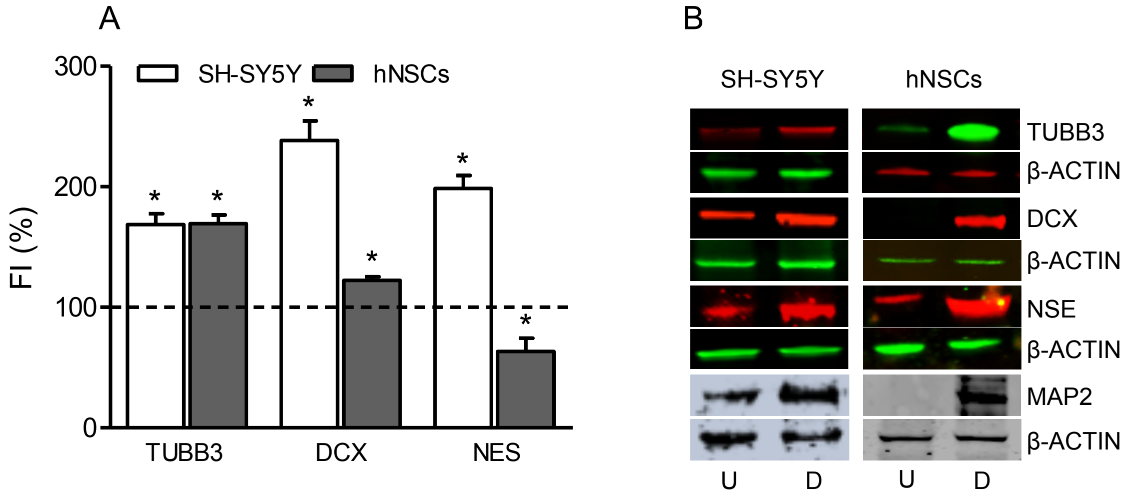

Figure S3. Markers of neuronal differentiation (TUBB3, DCX, NES, NSE and MAP2) determined by flow cytometry (A) and Western blot (B). FI, fluorescence intensity. Dashed line (100 %) represents the mean value of undifferentiated (U) cells. Bars indicate the mean and standard deviation values of differentiated (D) cells. β-ACTIN was used to correct for the protein loading. Independent experiments, N = 3 (A) and 2 (B). *, $p < 0.05$ (versus U cells, Mann-Whitney).

To confirm the dopaminergic phenotype, we determined tyrosine hydroxylase (TH) levels, which is the rate-limiting enzyme of dopamine synthesis [24], and dopamine transporter (DAT), the protein involved in the reuptake of extracellular dopamine into presynaptic neurons [25]. The levels of these two proteins are commonly used as dopaminergic differentiation markers [1,26-28]. FC analysis showed that the amount of both proteins increased with differentiation (Figure S4A). The FC results were confirmed by WB analysis (Figure S4B). Our differentiation protocol also increased the amount of intracellular dopamine (Figure S4C). Moreover, differentiated cells were electrically active neurons because they released dopamine after KCl stimulation (Figure S4D).

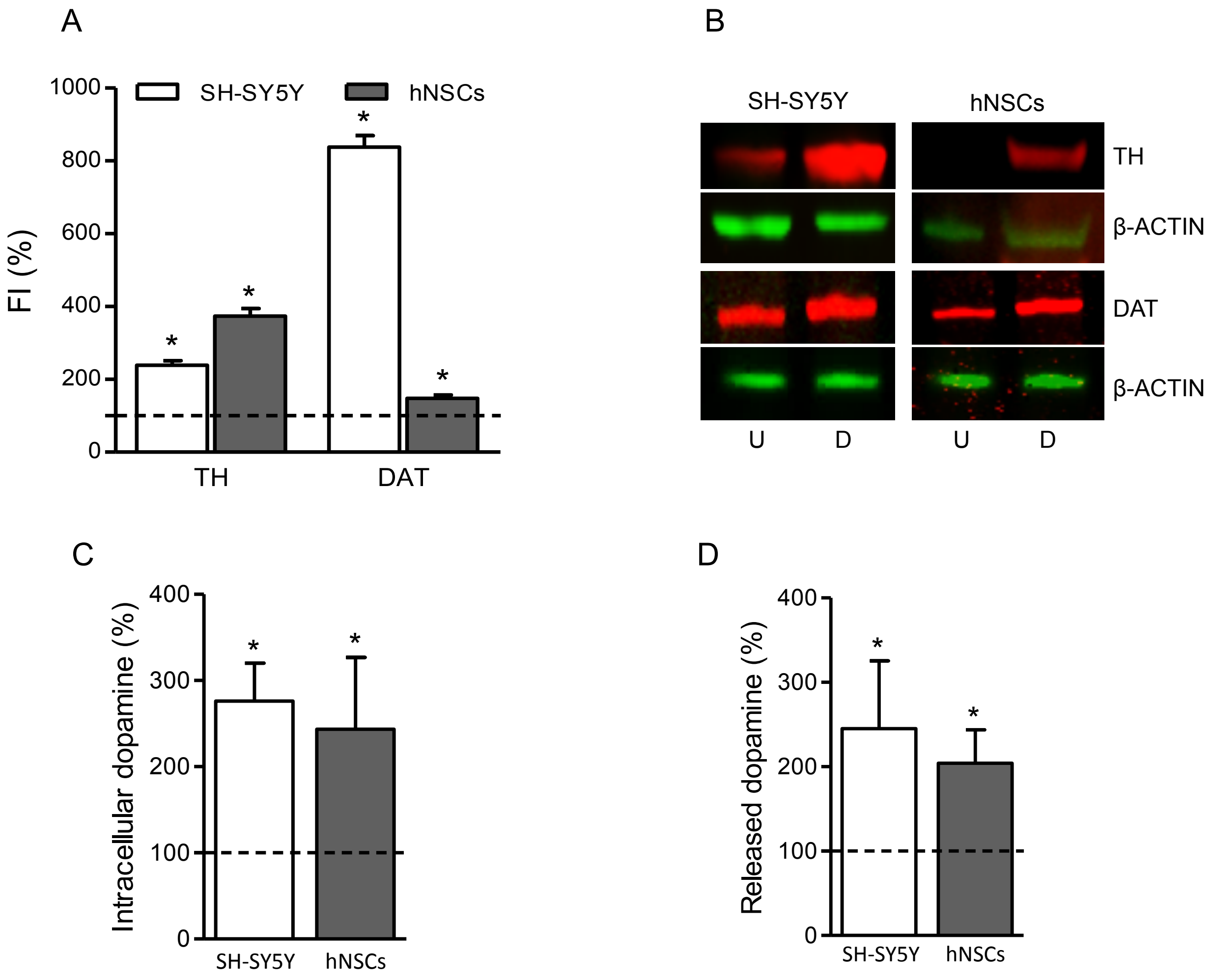

Figure S4. Markers of dopaminergic neuronal differentiation (TH, DAT, intracellular dopamine, and released dopamine) determined by flow cytometry (A), Western blot (B) or ELISA (C, D). FI, fluorescence intensity. Dashed lines (100 %) represent the mean values of undifferentiated (U) cells. Bars indicate the mean and standard deviation values of differentiated (D) cells. β-ACTIN was used to correct for the protein loading. Independent experiments, N = 3 (A, C, D) and 2 (B). *, $p < 0.05$ (versus U cells, Mann-Whitney).

To check the specificity of our differentiation protocol, we performed immunocytochemistry for neuronal (TUBB3) and dopaminergic (TH) markers and observed that most of the differentiated neurons stained for TH (Figure S5A). Confirming the specificity of dopaminergic neuronal differentiation, cells did not increase levels of intracellular acetylcholine or noradrenaline nor responded to KCl stimulation by releasing more noradrenaline (Figure S5B-D).

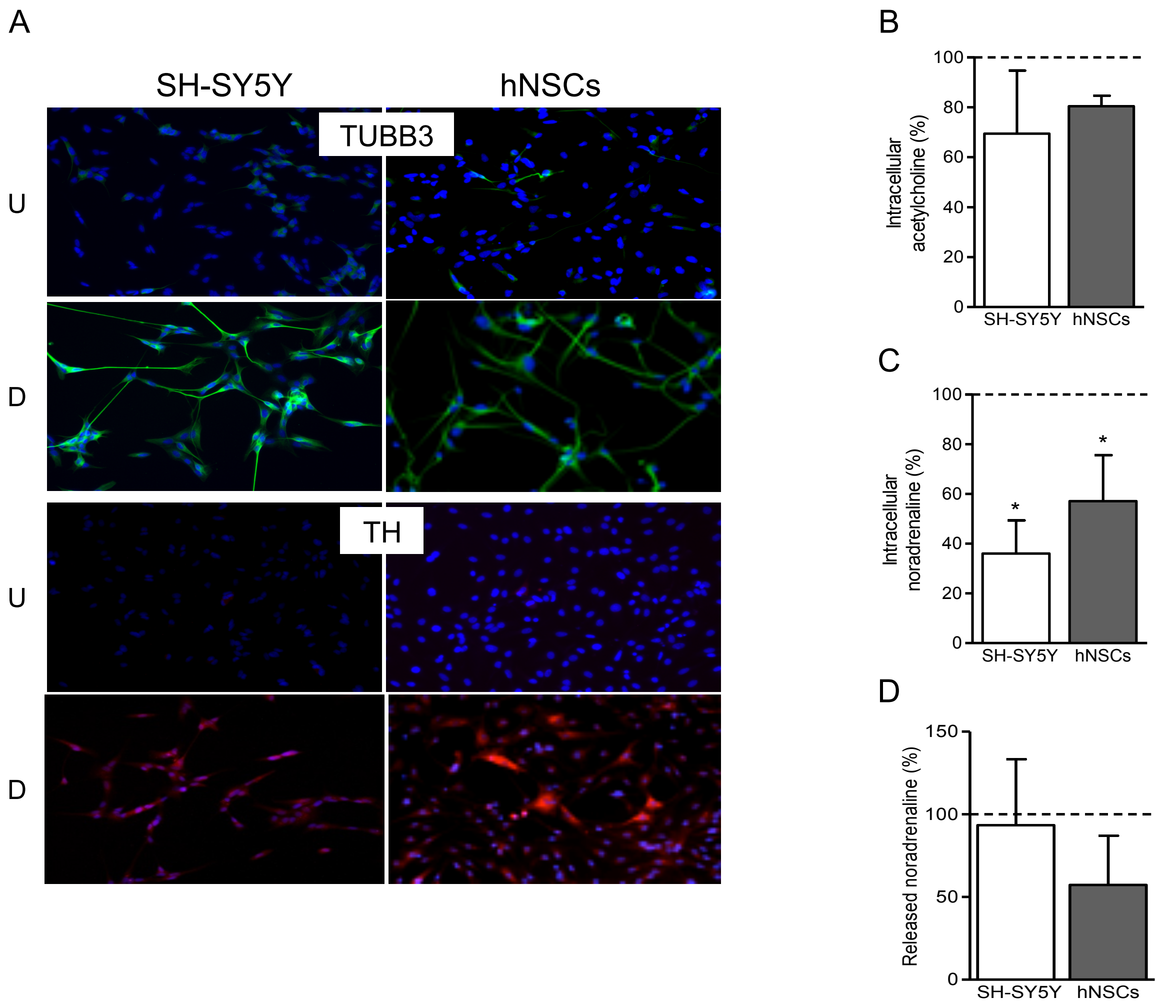

Figure S5. Specificity of dopaminergic neuronal differentiation. A) Representative TUBB3 (green) and TH (red) immunocytochemical images of neuroblastoma SH-SY5Y cells and hNSCs. U, undifferentiated cells. D, differentiated cells. B) Intracellular acetylcholine levels. C) Intracellular noradrenaline levels. D) Extracellular noradrenaline levels. Dashed lines (100 %) represent the mean values of U cells. Bars indicate the mean and standard deviation values of D cells. Independent experiments, N = 3 (B, C, D). *, $p < 0.05$ (versus U cells, Mann-Whitney).

All these results indicate that neuroblastoma SH-SY5Y cells can differentiate into dopaminergic neurons in similar ways to NSCs [26,29,30]. Therefore, these neuroblastoma cells are good models to study dopaminergic neuronal differentiation.

SUPPLEMENTAL NOTE 2- OXPHOS changes along dopaminergic neuronal differentiation

The peroxisome proliferator-activated receptor gamma (PPARG) has been associated to mitochondrial biogenesis [31]. mRNA levels for this factor significantly increased with the differentiation in the analyzed cells (Figure S6A).

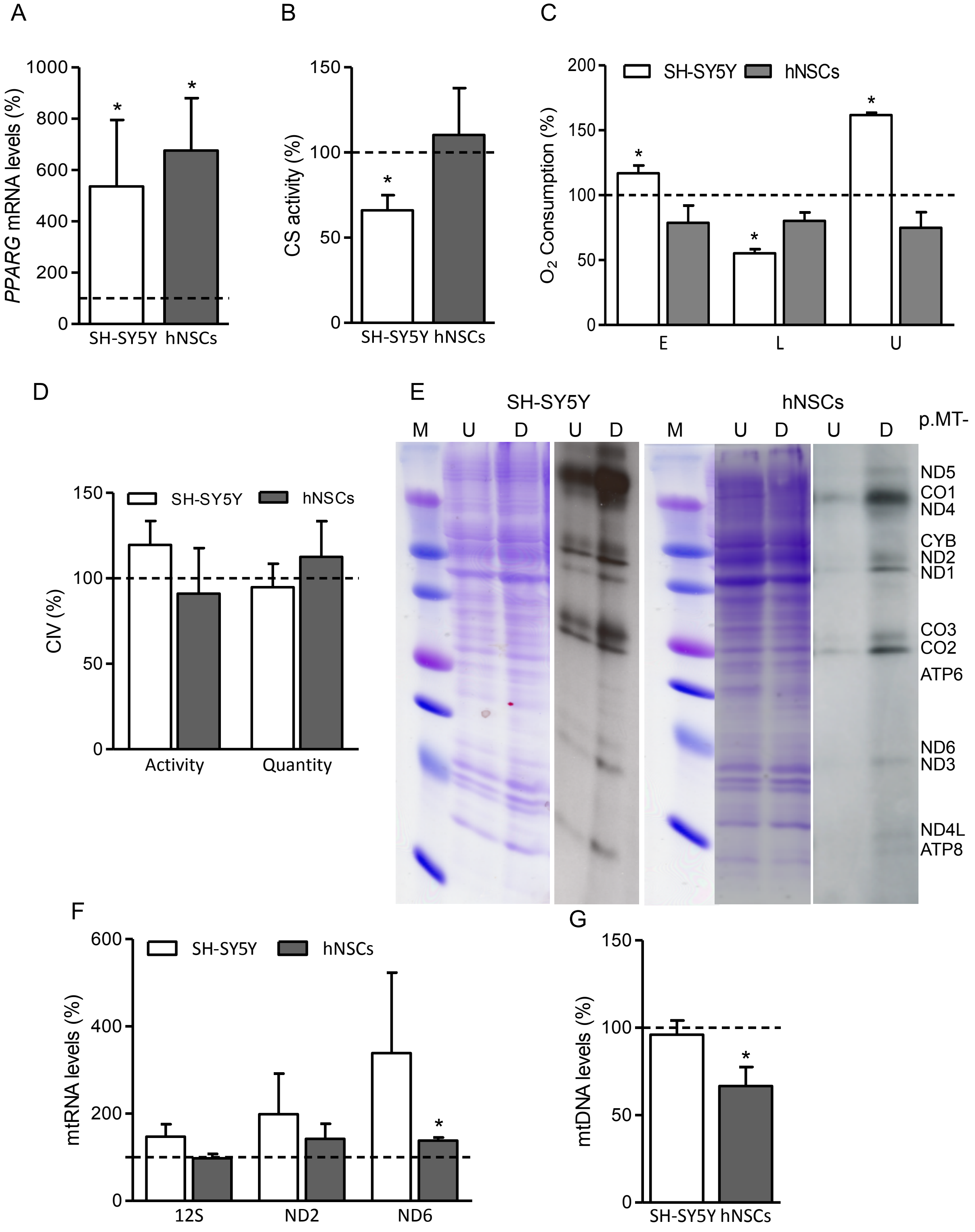

Figure S6. OXPHOS changes during dopaminergic neuronal differentiation. A) *PPARG* mRNA levels. Independent experiments, N = 3. B) Citrate synthase specific activity (CS sa). N = 3. C) Oxygen consumption. E, L and U code for endogenous, leaking and uncoupled respiration, respectively. N = 3. D) Complex IV (CIV) sa and quantity normalized by CS sa. N = 3. E) Representative image of gels for mitochondrial protein synthesis analysis. Gels showing the loading control and electrophoretic patterns of mitochondrial translation products are included. M, U and D code for molecular weight marker, undifferentiated and differentiated cells, respectively. p.MT-ND1-6 and p.MT-ND4L are CI mtDNA-encoded subunits; p.MT-CYB is a CIII mtDNA-encoded subunit; p.MT-CO1-3 are CIV mtDNA-encoded subunits; and p.MT-ATP6,8 are CV mtDNA-encoded subunits. N = 3 (SH-SY5Y) and 2 (hNSCs). F) Mitochondrial RNA levels. *12S* rRNA, *ND2* and *ND6* mRNAs. N = 4 (SH-SY5Y) and 3 (hNSCs). G) mtDNA levels. N = 3. Dashed lines (100 %) represent the mean values of U cells. Bars indicate the mean and standard deviation values of D cells, respectively. *, $p < 0.05$ (versus U cells, Mann-Whitney).

The specific activity of the mitochondrial matrix enzyme citrate synthase (CS) is usually used as a marker of mitochondrial volume or number. The absence of a significant difference in CS specific activity between differentiated and undifferentiated hNSCs suggested that the cell fraction occupied by mitochondria did not change with the cell differentiation (Figure S6B). On the contrary, differentiated SH-SY5Y cells showed lower CS specific activity than undifferentiated ones.

Endogenous and uncoupled oxygen consumption per cell increased with differentiation in the neuroblastoma cell line but it did not change in hNSCs (Figure S6C). Differentiated SH-SY5Y cells showed significantly lower leaking oxygen consumption than undifferentiated ones. CIV quantity and specific activity, normalized by CS specific activity, did not change in hNSCs or SH-SY5Y cells (Figure S6D). Mitochondrial protein synthesis in hNSCs and SH-SY5Y cells increased after differentiation (Figure S6E).

Mitochondrial transcript levels did not change with neuronal differentiation (Figure S6F). mtDNA amount significantly decreased in hNSCs but did not change in SH-SY5Y cells (Figure S6G).

SUPPLEMENTAL NOTE 3- OXPHOS-related mutant proteins affect OXPHOS function of neuroblastoma SH-SY5Y cells

The mtDNA is replicated by the mitochondrial DNA polymerase gamma (POLG). The POLG p.Y955C amino acid change has been associated to progressive external ophthalmoplegia (PEO) [32]. Cells overexpressing POLG showed higher levels of *POLG* mRNA and POLG protein than parental cells. The most abundant *POLG* mRNA derives from the transfected gene (Figure S7).

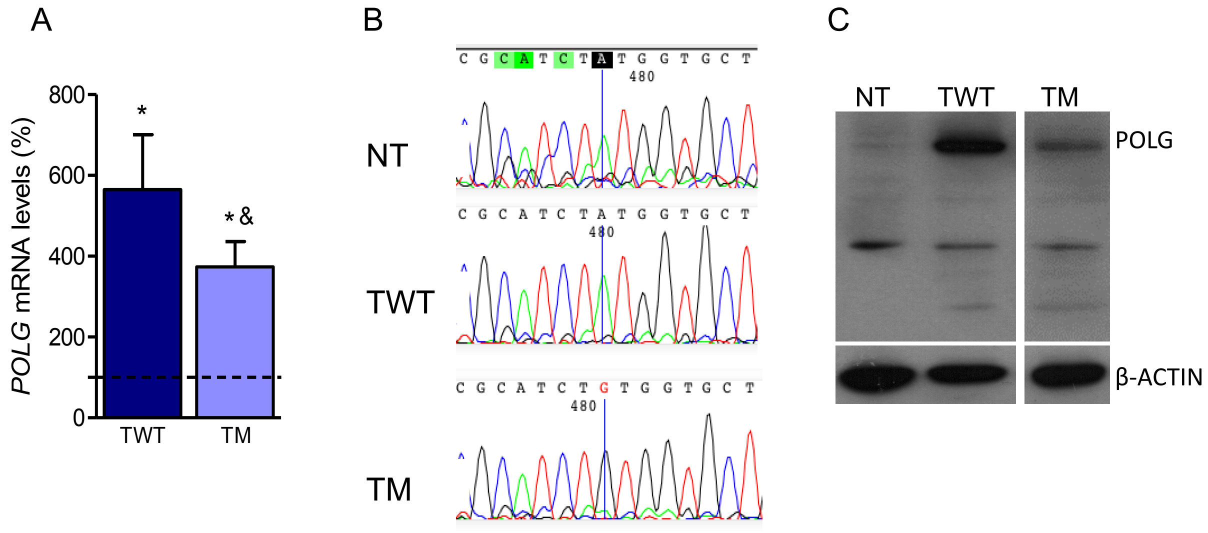

Figure S7. Overexpression of a mutant mitochondrial DNA polymerase gamma (POLG) in neuroblastoma SH-SY5Y cells. A) mRNA levels. Independent experiments, N = 3. B) cDNA sequence. C) protein levels. β-ACTIN was used to correct for the protein loading. NT, TWT and TM code for non-transfected cells and transfected with wild-type or mutant *POLG* sequences, respectively. Dashed line (100 %) represents the mean value of NT cells. Bars indicate the mean and standard deviation values of TWT and TM cells. *, $p < 0.05$ (vs. NT cells, Mann-Whitney). $^{\&}$, $p < 0.05$ (vs. TWT cells, Mann-Whitney).

Mitochondrial mRNAs are translated in mitochondrial ribosomes. The mitochondrial ribosomal protein S12 (MRPS12) is a critical component of the ribosomal decoding center [33]. As there were no pathologic mutations described in this gene, we performed an ortholog protein alignment to select an amino acid position that, after mutated, could affect mitochondrial translation. The 72-KPNS-75 amino acids were conserved in 100 % of 197 reference sequences from animals, plants and fungi (GenBank. Accessed in November 23, 2016). The primary amine at the end of the lysine side-chain at position 72, K43 in *Escherichia coli*, interacts and restricts the motion of aminoacyl-tRNA binding site (A-site) nucleotide A1557 in the mitochondrial *12S* rRNA, A1492 in *E.coli 16S* rRNA [34]. Thus, we generated the mutation MRPS12 p.K72L. This amino acid substitution removed hydrogen bindings and would be expected to make the assembly of a complex between A1557 with an A-site tRNA less probable, then originating a hyper-accuracy phenotype [34]. Cells overexpressing MRPS12 showed higher levels of *MRPS12* mRNA and MRPS12 protein than parental cells. The most abundant *MRPS12* mRNA derives from the transfected gene (Figure S8).

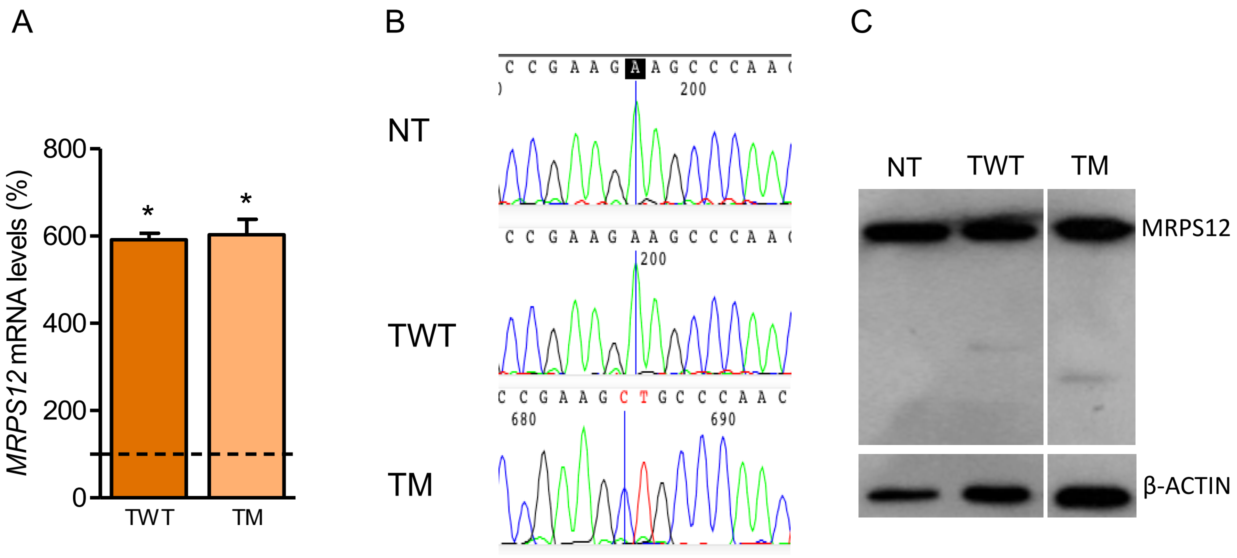

Figure S8. Overexpression of a mutant mitochondrial ribosomal protein S12 (MRPS12) in neuroblastoma SH-SY5Y cells. A) mRNA levels. Independent experiments, N = 2. B) cDNA sequence. C) protein levels. β-ACTIN was used to correct for the protein loading. NT, TWT and TM code for non-transfected cells and transfected with wild-type or mutant *MRPS12* sequences, respectively. Dashed line (100 %) represents the mean value of NT cells. Bars indicate the mean and standard deviation values TWT and TM cells. *, $p < 0.05$ (vs. NT cells, Mann-Whitney).

The Rieske iron sulfur protein (UQCRFS1) is a CIII key component. There are no pathologic mutations described in this gene. Then, we performed a protein alignment to select an amino acid position that, after mutated, could affect CIII activity. The 217-CTHLGC-222 amino acids were conserved in ≥ 99.6 % of 241 reference sequences from animals, plants, fungi and protists (GenBank. Accessed in November 22, 2016). The cysteine at position 222 is part of a disulfide bridge important for the complex activity [35]. Thus, we generated the mutation UQCRFS1 p.C222S. This substitution, C138S in *Rhodobacter capsulatus* [36], C134S in *Rhodobacter sphaeroides* [37] and C164S in *Sacharomyces cerevisiae* [38], provokes a reduction in CIII activity. Cells overexpressing UQCRFS1 showed higher levels of *UQCRFS1* mRNA and UQCRFS1 protein than parental cells. The most abundant *UQCRFS1* mRNA sequence derives from the transfected gene (Figure S9).

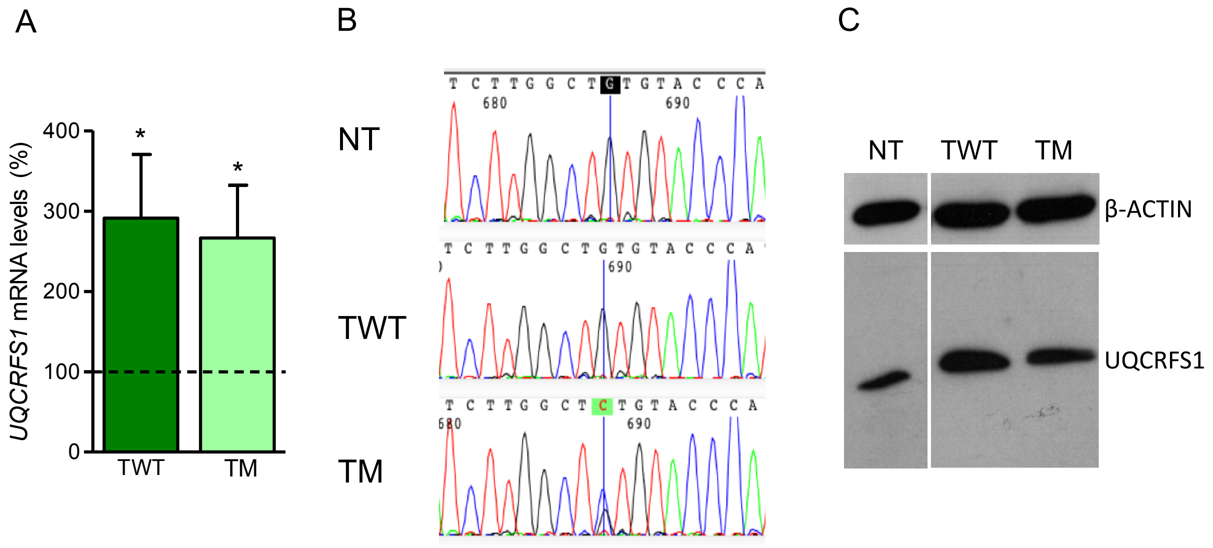

Figure S9. Overexpression of a mutant Rieske iron sulfur protein (UQCRFS1) in neuroblastoma SH-SY5Y cells. A) mRNA levels. Independent experiments, N = 6. B) cDNA sequence. C) protein levels. β-ACTIN was used to correct for the protein loading. NT, TWT and TM code for non-transfected cells and transfected with wild-type or mutant *UQCRFS1* sequences, respectively. Dashed line (100 %) represents the mean value of NT cells. Bars indicate the mean and standard deviation values of TWT and TM cells. *, $p < 0.05$ (vs. NT cells, Mann-Whitney).

SUPPLEMENTAL NOTE 4- Neuroblastoma SH-SY5Y rho^0 cell line

We have determined the nuclear genetic fingerprint of neuroblastoma SH-SY5Y rho^0 cells and found that match 15 out of 16 markers of the parental rho$^+$ cells (Table S1). They have lost the allele 17 for the D2S1338 marker. However, loss of heterozygosity is a frequent genetic alteration in cancer cell lines [39]. On the other hand, the rho^0 cell line, obtained by treatment of parental rho$^+$ cell line with the DNA intercalating agent ethidium bromide, was almost tetraploid (chromosomal modal number, 89; range 80-89) (Figure S10A). This kind of aneuploidy has already been reported for cybrids derived from other rho^0 cell lines [40].

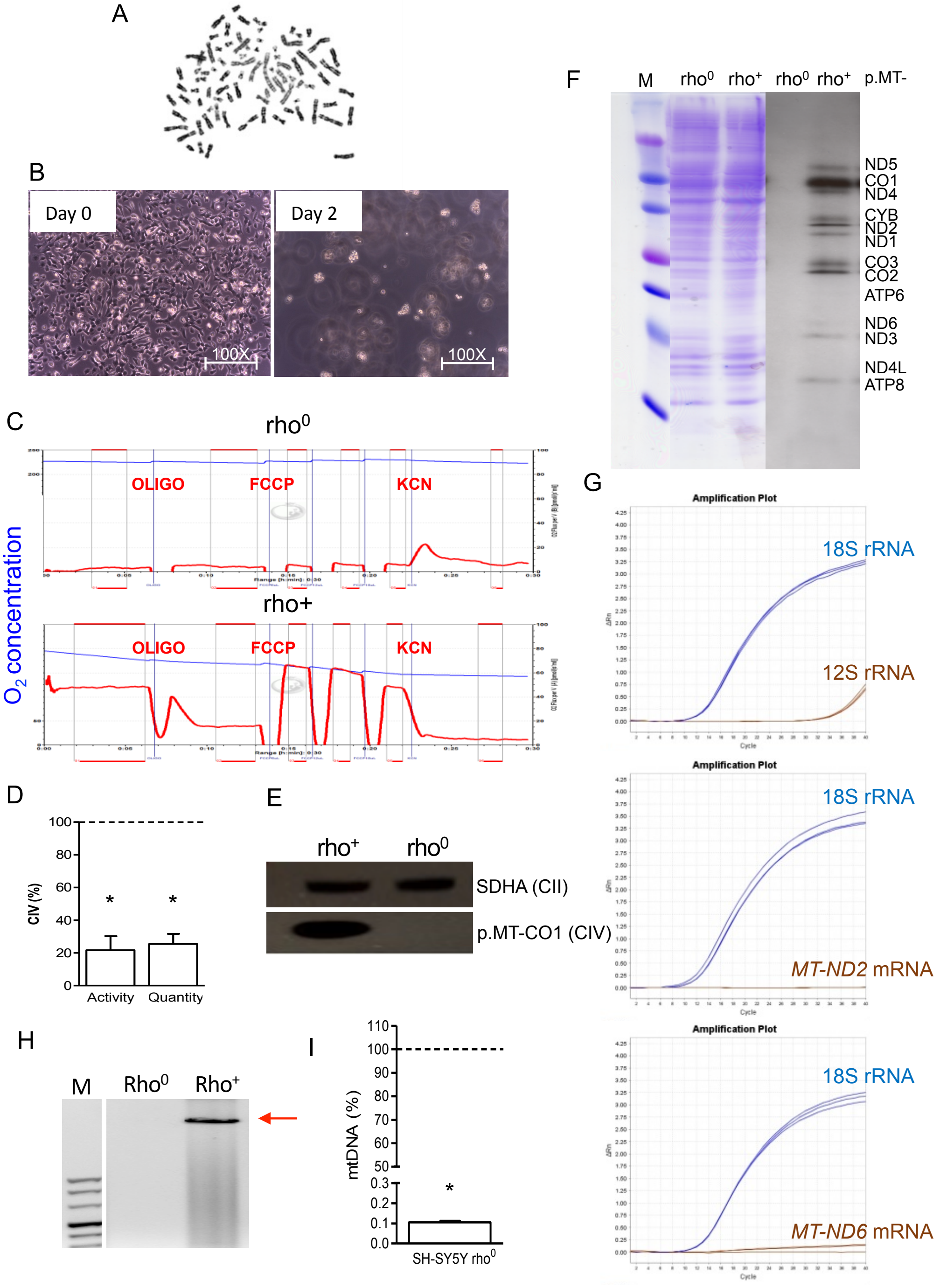

Figure S10. Neuroblastoma SH-SY5Y rho^0 cells. A) Karyotype. B) Representative pictures of rho^0 cells growing in 5 mM galactose. C) Oxygen consumption. OLIGO, FCCP and KCN code for oligomycin, carbonyl cyanide-4-(trifluoromethoxy)phenylhydrazone, and potassium cyanide, respectively. Independent experiments, N = 2. D) CIV specific activity and quantity. N = 3. E) CIV p.MT-CO1 levels. SDHA is an nDNA-encoded CII subunit. F) Mitochondrial protein synthesis. Gels showing the loading control and the electrophoretic patterns of mitochondrial translation products are included. M, molecular weight marker. p.MT-ND1-6 and p.MT-ND4L are CI mtDNA-encoded subunits; p.MT-CYB is a CIII mtDNA-encoded subunit; p.MT-CO1-3 are CIV mtDNA-encoded subunits; and p.MT-ATP6,8 are CV mtDNA-encoded subunits. N = 2. G) mtDNA-encoded RNA levels (brown lines). The nDNA-encoded *18S* rRNA is represented as blue lines. N = 2. H) mtDNA amplicon (red arrow). M, molecular weigh marker. H) mtDNA levels. N = 3. Dashed lines (100 %) represent the mean values of rho$^+$ parental cells. Bars indicate the mean and standard deviation values of rho^0 cells. *, $p < 0.05$ (versus rho$^+$ parental cells, Mann-Whitney).

This neuroblastoma SH-SY5Y rho^0 cell line dies when grown at 5 mM galactose (Figure S10B), does not consume oxygen nor respond to OXPHOS inhibitors or uncouplers (Figure S10C), has a residual CIV activity and quantity (Figure S10D), does not synthesize mtDNA-encoded polypeptides (Figure S10E, F), nor produces RNA from mtDNA genes (Figure S10G), and neither has mtDNA, as confirmed by PCR electrophoresis or qPCR (Figure S10H, I).

SUPPLEMENTAL NOTE 5- Effect of prenatal exposure to linezolid on mouse brain

The linezolid (LIN) effect on brain development was tested using C57BL/6J mice. Vehicle, 5 mg LIN, 70 mg uridine or 5 mg LIN plus 70 mg uridine were daily provided, by oral gavage, to pregnant mice during embryonic days E8 and E15. In E16, mothers were sacrificed and different parameters were determined in the fetal brains (Figure S11). There were no significant differences between groups.

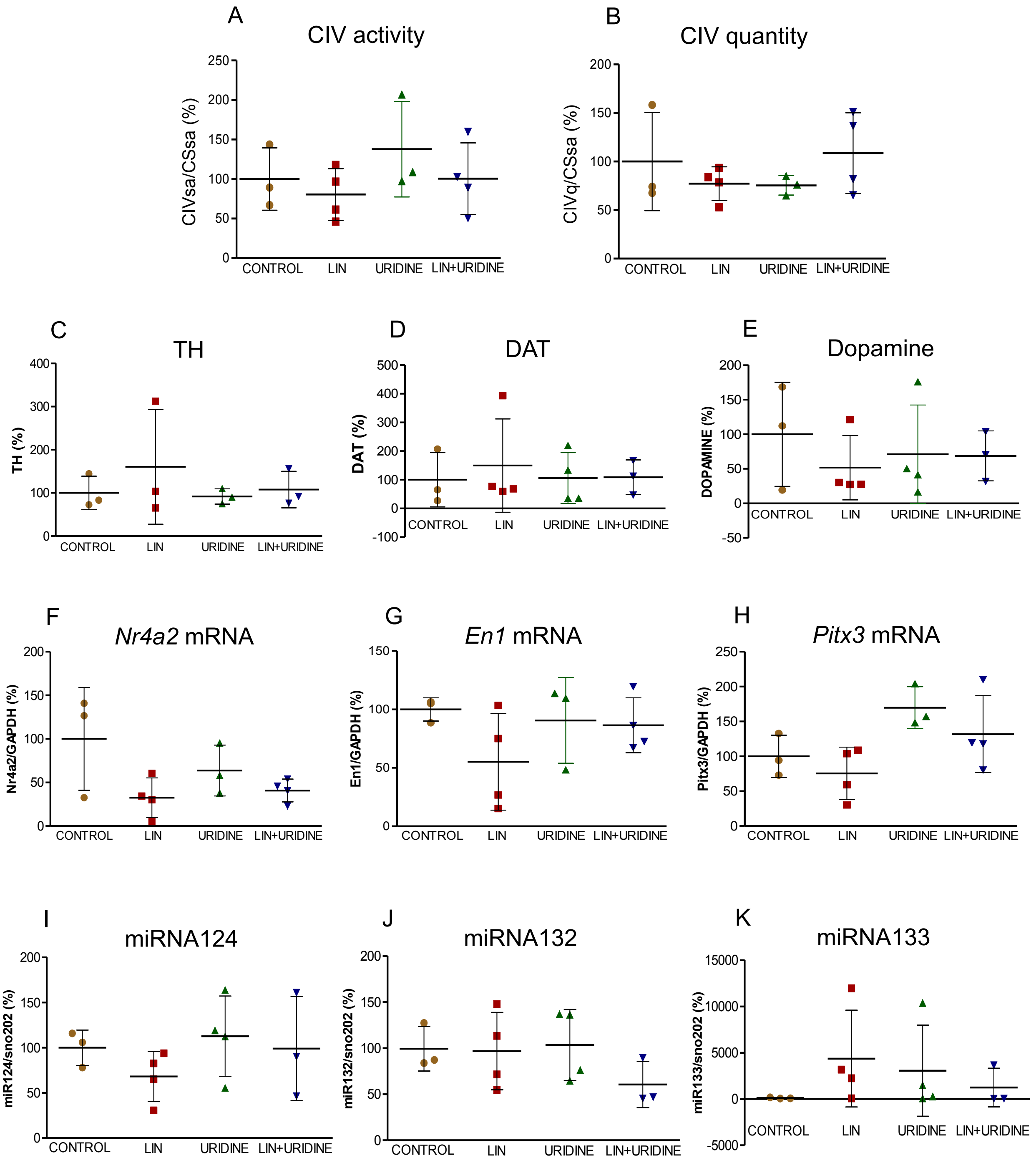

Figure S11. Levels of different brain parameters from mice prenatally exposed to linezolid, uridine or linezolid plus uridine. Independent experiments, N ≥ 3. A, B) CIV specific activity and quantity, normalized by CS specific activity. C-E) Levels of dopaminergic neuronal markers (TH, DAT and dopamine). F-H) mRNA levels of genes required for dopaminergic neuronal differentiation, normalized by *GAPDH* mRNA amount. I-K) Levels of different miRNAs related with dopaminergic neuronal differentiation, normalized by sno202. Points represent individual samples and horizontal lines indicate mean ± standard deviation values.

REFERENCES

1. Xie, H.R.; Hu, L.S.; Li, G.Y. SH-SY5Y human neuroblastoma cell line: in vitro cell model of dopaminergic neurons in Parkinson's disease. *Chin Med J (Engl)* **2010**, *123*, 1086-1092.
2. Xicoy, H.; Wieringa, B.; Martens, G.J. The SH-SY5Y cell line in Parkinson's disease research: a systematic review. *Mol Neurodegener* **2017**, *12*, 10, doi:10.1186/s13024-017-0149-0 10.1186/s13024-017-0149-0 [pii].
3. Yu, M.; Selvaraj, S.K.; Liang-Chu, M.M.; Aghajani, S.; Busse, M.; Yuan, J.; Lee, G.; Peale, F.; Klijn, C.; Bourgon, R., et al. A resource for cell line authentication, annotation and quality control. *Nature* **2015**, *520*, 307-311, doi:10.1038/nature14397 nature14397 [pii].
4. Jiang, T.; Wang, H. Identification of cross-contamination in SH-SY5Y cell line. *Hum Cell* **2014**, *27*, 176-178, doi:10.1007/s13577-014-0096-9.
5. Mamaeva, S.E. Karyotypic evolution of cells in culture: a new concept. *Int Rev Cytol* **1998**, *178*, 1-40.
6. Pahlman, S.; Mamaeva, S.; Meyerson, G.; Mattsson, M.E.; Bjelfman, C.; Ortoft, E.; Hammerling, U. Human neuroblastoma cells in culture: a model for neuronal cell differentiation and function. *Acta Physiol Scand Suppl* **1990**, *592*, 25-37.
7. Kim, G.J.; Park, S.Y.; Kim, H.; Chun, Y.H.; Park, S.H. Chromosomal aberrations in neuroblastoma cell lines identified by cross species color banding and chromosome painting. *Cancer Genet Cytogenet* **2001**, *129*, 10-16, doi:S0165-4608(01)00418-6 [pii].
8. Spengler, B.A.; Biedler, J.L.; Ross, R.A. A corrected karyotype for the SH-SY5Y human neuroblastoma cell line. *Cancer Genet Cytogenet* **2002**, *138*, 177-178, doi:S016546080200523X [pii].
9. Cohen, N.; Betts, D.R.; Rechavi, G.; Amariglio, N.; Trakhtenbrot, L. Clonal expansion and not cell interconversion is the basis for the neuroblast and nonneuronal types of the SK-N-SH neuroblastoma cell line. *Cancer Genet Cytogenet* **2003**, *143*, 80-84, doi:S016546080200835X [pii].
10. Yusuf, M.; Leung, K.; Morris, K.J.; Volpi, E.V. Comprehensive cytogenomic profile of the in vitro neuronal model SH-SY5Y. *Neurogenetics* **2013**, *14*, 63-70, doi:10.1007/s10048-012-0350-9.
11. Khan, F.H.; Pandian, V.; Ramraj, S.; Natarajan, M.; Aravindan, S.; Herman, T.S.; Aravindan, N. Acquired genetic alterations in tumor cells dictate the development of high-risk neuroblastoma and clinical outcomes. *BMC Cancer* **2015**, *15*, 514, doi:10.1186/s12885-015-1463-y 10.1186/s12885-015-1463-y [pii].
12. Ly, P.; Eskiocak, U.; Kim, S.B.; Roig, A.I.; Hight, S.K.; Lulla, D.R.; Zou, Y.S.; Batten, K.; Wright, W.E.; Shay, J.W. Characterization of aneuploid populations with trisomy 7 and 20 derived from diploid human colonic epithelial cells. *Neoplasia* **2011**, *13*, 348-357.
13. van Oven, M.; Kayser, M. Updated comprehensive phylogenetic tree of global human mitochondrial DNA variation. *Hum Mutat* **2009**, *30*, E386-394, doi:10.1002/humu.20921.
14. Khusnutdinova, E.; Gilyazova, I.; Ruiz-Pesini, E.; Derbeneva, O.; Khusainova, R.; Khidiyatova, I.; Magzhanov, R.; Wallace, D.C. A mitochondrial etiology of neurodegenerative diseases: evidence from Parkinson's disease. *Ann N Y Acad Sci* **2008**, *1147*, 1-20, doi:10.1196/annals.1427.001 NYAS1147001 [pii].
15. Miller, S.W.; Trimmer, P.A.; Parker, W.D., Jr.; Davis, R.E. Creation and characterization of mitochondrial DNA-depleted cell lines with "neuronal-like"

properties. *J Neurochem* **1996**, *67*, 1897-1907.
16. Pereira, L.; Soares, P.; Radivojac, P.; Li, B.; Samuels, D.C. Comparing phylogeny and the predicted pathogenicity of protein variations reveals equal purifying selection across the global human mtDNA diversity. *Am J Hum Genet* **2011**, *88*, 433-439, doi:10.1016/j.ajhg.2011.03.006 S0002-9297(11)00098-X [pii].
17. Martin-Navarro, A.; Gaudioso-Simon, A.; Alvarez-Jarreta, J.; Montoya, J.; Mayordomo, E.; Ruiz-Pesini, E. Machine learning classifier for identification of damaging missense mutations exclusive to human mitochondrial DNA-encoded polypeptides. *BMC Bioinformatics* **2017**, *18*, 158, doi:10.1186/s12859-017-1562-7 10.1186/s12859-017-1562-7 [pii].
18. Krishna, A.; Biryukov, M.; Trefois, C.; Antony, P.M.; Hussong, R.; Lin, J.; Heinaniemi, M.; Glusman, G.; Koglsberger, S.; Boyd, O., et al. Systems genomics evaluation of the SH-SY5Y neuroblastoma cell line as a model for Parkinson's disease. *BMC Genomics* **2014**, *15*, 1154, doi:10.1186/1471-2164-15-1154 1471-2164-15-1154 [pii].
19. Heraud, C.; Hilairet, S.; Muller, J.M.; Leterrier, J.F.; Chadeneau, C. Neuritogenesis induced by vasoactive intestinal peptide, pituitary adenylate cyclase-activating polypeptide, and peptide histidine methionine in SH-SY5y cells is associated with regulated expression of cytoskeleton mRNAs and proteins. *J Neurosci Res* **2004**, *75*, 320-329, doi:10.1002/jnr.10866.
20. Bazan, E.; Alonso, F.J.; Redondo, C.; Lopez-Toledano, M.A.; Alfaro, J.M.; Reimers, D.; Herranz, A.S.; Paino, C.L.; Serrano, A.B.; Cobacho, N., et al. In vitro and in vivo characterization of neural stem cells. *Histol Histopathol* **2004**, *19*, 1261-1275, doi:10.14670/HH-19.1261.
21. Kaiser, E.; Kuzmits, R.; Pregant, P.; Burghuber, O.; Worofka, W. Clinical biochemistry of neuron specific enolase. *Clin Chim Acta* **1989**, *183*, 13-31, doi:0009-8981(89)90268-4 [pii].
22. Lopes, F.M.; Schroder, R.; da Frota, M.L., Jr.; Zanotto-Filho, A.; Muller, C.B.; Pires, A.S.; Meurer, R.T.; Colpo, G.D.; Gelain, D.P.; Kapczinski, F., et al. Comparison between proliferative and neuron-like SH-SY5Y cells as an in vitro model for Parkinson disease studies. *Brain Res* **2010**, *1337*, 85-94, doi:10.1016/j.brainres.2010.03.102 S0006-8993(10)00765-1 [pii].
23. Pezzini, F.; Bettinetti, L.; Di Leva, F.; Bianchi, M.; Zoratti, E.; Carrozzo, R.; Santorelli, F.M.; Delledonne, M.; Lalowski, M.; Simonati, A. Transcriptomic profiling discloses molecular and cellular events related to neuronal differentiation in SH-SY5Y neuroblastoma cells. *Cell Mol Neurobiol* **2017**, *37*, 665-682, doi:10.1007/s10571-016-0403-y 10.1007/s10571-016-0403-y [pii].
24. Daubner, S.C.; Le, T.; Wang, S. Tyrosine hydroxylase and regulation of dopamine synthesis. *Arch Biochem Biophys* **2011**, *508*, 1-12, doi:10.1016/j.abb.2010.12.017 S0003-9861(10)00514-X [pii].
25. Vaughan, R.A.; Foster, J.D. Mechanisms of dopamine transporter regulation in normal and disease states. *Trends Pharmacol Sci* **2013**, *34*, 489-496, doi:10.1016/j.tips.2013.07.005 S0165-6147(13)00134-X [pii].
26. Swistowski, A.; Peng, J.; Han, Y.; Swistowska, A.M.; Rao, M.S.; Zeng, X. Xeno-free defined conditions for culture of human embryonic stem cells, neural stem cells and dopaminergic neurons derived from them. *PLoS One* **2009**, *4*, e6233, doi:10.1371/journal.pone.0006233.
27. Kovalevich, J.; Langford, D. Considerations for the use of SH-SY5Y neuroblastoma cells in neurobiology. *Methods Mol Biol* **2013**, *1078*, 9-21, doi:10.1007/978-1-62703-640-5_2.

28. Chen, C.; Ma, Q.; Chen, X.; Zhong, M.; Deng, P.; Zhu, G.; Zhang, Y.; Zhang, L.; Yang, Z.; Zhang, K., et al. Thyroid hormone-Otx2 signaling is required for embryonic ventral midbrain neural stem cells differentiated into dopamine neurons. *Stem Cells Dev* **2015**, *24*, 1751-1765, doi:10.1089/scd.2014.0489.
29. Bennett, L.; Yang, M.; Enikolopov, G.; Iacovitti, L. Circumventricular organs: a novel site of neural stem cells in the adult brain. *Mol Cell Neurosci* **2009**, *41*, 337-347, doi:10.1016/j.mcn.2009.04.007 S1044-7431(09)00085-2 [pii].
30. Wang, W.; Osenbroch, P.; Skinnes, R.; Esbensen, Y.; Bjoras, M.; Eide, L. Mitochondrial DNA integrity is essential for mitochondrial maturation during differentiation of neural stem cells. *Stem Cells* **2010**, *28*, 2195-2204, doi:10.1002/stem.542.
31. Miglio, G.; Rosa, A.C.; Rattazzi, L.; Collino, M.; Lombardi, G.; Fantozzi, R. PPARgamma stimulation promotes mitochondrial biogenesis and prevents glucose deprivation-induced neuronal cell loss. *Neurochem Int* **2009**, *55*, 496-504, doi:10.1016/j.neuint.2009.05.001 S0197-0186(09)00161-2 [pii].
32. Van Goethem, G.; Dermaut, B.; Lofgren, A.; Martin, J.J.; Van Broeckhoven, C. Mutation of POLG is associated with progressive external ophthalmoplegia characterized by mtDNA deletions. *Nat Genet* **2001**, *28*, 211-212, doi:10.1038/90034 90034 [pii].
33. Emperador, S.; Pacheu-Grau, D.; Bayona-Bafaluy, M.P.; Garrido-Perez, N.; Martin-Navarro, A.; Lopez-Perez, M.J.; Montoya, J.; Ruiz-Pesini, E. An MRPS12 mutation modifies aminoglycoside sensitivity caused by 12S rRNA mutations. *Front Genet* **2014**, *5*, 469, doi:10.3389/fgene.2014.00469.
34. Panecka, J.; Mura, C.; Trylska, J. Interplay of the bacterial ribosomal A-site, S12 protein mutations and paromomycin binding: a molecular dynamics study. *PLoS One* **2014**, *9*, e111811, doi:10.1371/journal.pone.0111811 PONE-D-14-20278 [pii].
35. Merbitz-Zahradnik, T.; Zwicker, K.; Nett, J.H.; Link, T.A.; Trumpower, B.L. Elimination of the disulfide bridge in the Rieske iron-sulfur protein allows assembly of the [2Fe-2S] cluster into the Rieske protein but damages the ubiquinol oxidation site in the cytochrome bc1 complex. *Biochemistry* **2003**, *42*, 13637-13645, doi:10.1021/bi035344r.
36. Davidson, E.; Ohnishi, T.; Tokito, M.; Daldal, F. Rhodobacter capsulatus mutants lacking the Rieske FeS protein form a stable cytochrome bc1 subcomplex with an intact quinone reduction site. *Biochemistry* **1992**, *31*, 3351-3358.
37. Van Doren, S.R.; Gennis, R.B.; Barquera, B.; Crofts, A.R. Site-directed mutations of conserved residues of the Rieske iron-sulfur subunit of the cytochrome bc1 complex of Rhodobacter sphaeroides blocking or impairing quinol oxidation. *Biochemistry* **1993**, *32*, 8083-8091.
38. Graham, L.A.; Trumpower, B.L. Mutational analysis of the mitochondrial Rieske iron-sulfur protein of Saccharomyces cerevisiae. III. Import, protease processing, and assembly into the cytochrome bc1 complex of iron-sulfur protein lacking the iron-sulfur cluster. *J Biol Chem* **1991**, *266*, 22485-22492.
39. Bady, P.; Diserens, A.C.; Castella, V.; Kalt, S.; Heinimann, K.; Hamou, M.F.; Delorenzi, M.; Hegi, M.E. DNA fingerprinting of glioma cell lines and considerations on similarity measurements. *Neuro Oncol* **2012**, *14*, 701-711, doi:10.1093/neuonc/nos072 nos072 [pii].
40. Hao, H.; Morrison, L.E.; Moraes, C.T. Suppression of a mitochondrial tRNA gene mutation phenotype associated with changes in the nuclear background. *Hum Mol Genet* **1999**, *8*, 1117-1124, doi:ddc128 [pii].
